# Investigating the follow-up discontinuation among people living with HIV in North Shoa Zone, Oromia, Ethiopia

**DOI:** 10.3389/fpubh.2024.1436905

**Published:** 2024-10-24

**Authors:** Abebe Feyissa Amhare, Girma Garedew Goyomsa, Yasmeen Moshtaq A. Al Issa

**Affiliations:** ^1^Department of Public Health, College of Health Science, Salale University, Fitche, Ethiopia; ^2^School of Public Health, Xi’an Jiaotong University Health Science Center, Xi’an, China; ^3^Department of Psychology and Behavioral Sciences, Zhejiang University, Hangzhou, China

**Keywords:** COVID-19, HIV, people living with HIV, follow-up discontinuation, Ethiopia

## Abstract

**Introduction:**

Follow-up discontinuation among people living with HIV can increase mortality and HIV spread within communities. This study investigates the impact of the COVID-19 on follow-up discontinuation among people living with HIV in Ethiopia.

**Methods:**

This longitudinal study used retrospective document review to compare follow-up status of people living with HIV during the COVID-19 pandemic with their status from 2017 to 2019. Data from selected health facilities were collected using a checklist, entered and cleaned in Excel, and analyzed in Stata. Descriptive statistics were presented in tables and line graphs. Incidence risk (IR) and incidence rate ratios (IRR) were calculated.

**Results:**

Between 2017 and 2021, a total of 7,447 people living with HIV were registered to begin ART at selected health facilities. Annual retention rates were consistent from 2017 to 2019, ranging from 0.941 to 0.949. During the COVID-19 pandemic, they dropped to 0.837 in 2020 and 0.840 in 2021. Retention rates were significantly correlated with loss to follow-up (r: −0.959, *p* ≤ 0.001), death (r: −0.968, *p* ≤ 0.001), and transfer-out (r: −0.979, *p* ≤ 0.001). Moreover, the incidence of loss to follow-up (IRR: 3.00, 95%CI: 2.71–3.33, *p* ≤ 0.001), death (IRR: 3.61, 95%CI: 3.13–4.16, *p* ≤ 0.001), poor adherence (IRR: 2.27, 95%CI: 2.14–2.40, *p* ≤ 0.001), and severe malnutrition (IRR: 2.32, 95%CI: 2.18–2.47, *p* ≤ 0.001) significantly increased during the COVID-19.

**Conclusion:**

The study found that COVID-19 healthcare disruptions increased follow-up loss among people living with HIV and heightening the disease burden in Ethiopia.

## 1 Introduction

The outbreak of Coronavirus Disease 2019 (COVID-19) quickly developed into a global public health crisis, significantly impacting millions of lives worldwide (1, 2). Since the onset of the pandemic, the majority of deaths have occurred among individuals with chronic diseases (3). People living with HIV have been particularly vulnerable, both medically and socially, throughout the COVID-19 pandemic (4, 5).

HIV/AIDS-related services have faced severe disruptions due to the pandemic, with regular healthcare for individuals living with HIV being interrupted as resources shifted towards COVID-19 prevention. Consequently, many individuals missed appointments for care and antiretroviral therapy (ART), while some discontinued follow-up care altogether due to their heightened vulnerability to COVID-19. Health facilities providing HIV prevention, treatment, and care had to modify their operational modalities to cope with the challenges presented by the pandemic, often prioritizing COVID-19 patients over HIV care. This shift has led to delays in linking individuals to necessary HIV services, adversely affecting patient engagement in care (6). Additionally, many patients avoided seeking healthcare, even for urgent needs, due to fears of COVID-19 exposure (7–9). Interruptions in ART can result from various factors, including loss to follow-up, defaulting, or transferring out, all of which contribute to increased attrition and poorer health outcomes (10–13).

Discontinuation of follow-up care significantly impairs ART adherence, which is critical for achieving viral suppression (14). Higher adherence rates are linked to improved health outcomes, while lower adherence can lead to adverse physical and mental health consequences (6). Patients who remain engaged in care are more likely to adhere to ART, experience better health outcomes, and reduce the risk of HIV transmission to others (15). Disruptions in ART adherence and routine healthcare schedules may consequently diminish viral suppression rates among people living with HIV. Furthermore, such discontinuation poses significant challenges to meeting the UNAIDS 95–95-95 targets aimed at ending the AIDS epidemic by 2030 (16). Inconsistent engagement in care hampers individuals’ ability to know their HIV status, receive sustained treatment, and achieve viral suppression. Missed appointments and therapy interruptions can lead to decreased ART coverage, impacting the third target, as consistent adherence is essential for achieving viral suppression. Thus, follow-up discontinuation not only heightens health risks for individuals but also presents substantial obstacles to achieving these global goals by 2030. Retention in care is critical for both the health of individuals living with HIV and broader public health efforts (17–19). Discontinuation of follow-up or missed medical appointments are independently associated with a higher risk of HIV-related morbidity and mortality (20). The ability of individuals living with HIV to consistently attend in-person appointments is crucial for effective retention in care, a challenge exacerbated by the COVID-19 pandemic. The interruption of medical follow-up for people living with HIV has been reported in various countries, with findings indicating compromised medical care and psychosocial well-being during the pandemic (21, 22). In Ethiopia, the withdrawal of HIV-infected individuals has been a significant challenge, even before the pandemic (12, 13, 23, 24). Although the country has made notable progress toward the UNAIDS 95–95-95 targets, the COVID-19 outbreak may have further exacerbated these challenges. Despite this, comprehensive scientific evidence on the extent of interruptions in medical follow-up among people living with HIV during the pandemic in Ethiopia remains lacking. Understanding the magnitude of follow-up discontinuation is crucial, as it can lead to increased morbidity, mortality, drug resistance, and other complications. This study aims to assess follow-up discontinuation and its impact on the lives of people living with HIV during the COVID-19 pandemic in the North Shoa Zone, Oromia, Ethiopia. The findings will assist government efforts to address follow-up discontinuation and prepare for future public health emergencies.

## 2 Materials and methods

### 2.1 Study area and source of data

This study was conducted in selected public hospitals and health centers within the North Shoa Zone of the Oromia regional state, central Ethiopia, providing healthcare for HIV patients from January 2017 to December 2021. The zone is situated in the northwest part of Ethiopia, with its capital city, Fitche, located approximately 112 km from Addis Ababa, the capital city of Ethiopia. According to the 2007 Census conducted by the Central Statistical Agency of Ethiopia (CSA), the zone has a total population of 1,431,305, comprising 717,552 men and 713,753 women, and covers an area of 10,322.48 square kilometers.

### 2.2 Study design and sample

A longitudinal study design with retrospective document review was undertaken to compare the follow-up status of people living with HIV during the COVID-19 pandemic with their follow-up status from 2017 to 2019. To select study sites, we employed a random selection process among the 16 ART facilities in the North Shoa Zone. A complete list of these sites was generated, with each site assigned a unique identifier. Utilizing a random number generator, we selected four sites, ensuring equal probability of selection. We included all medical records of individuals documented as living with HIV from the selected health institutions, focusing solely on complete documents adhering to HIV follow-up standards and confirmed HIV-positive records. This sampling strategy allowed us to capture a diverse patient population, strengthening the validity of our findings by ensuring that the selected sites represented a cross-section of the healthcare landscape in the region.

### 2.3 Study materials and variables

We utilized the standard HIV CARE/ART FOLLOW-UP form utilized by ART clinics in Ethiopia. From this follow-up chart, we developed tools to guide us in extracting information. The following variables were extracted from these tools: Socio-demographic variables including age, sex, weight, height, and BMI; Functional status (Working, Ambulatory, Bedridden); HIV treatment-related variables such as Date of ART initiation, Regimen line, WHO Stage, Number of new HIV infections, ART adherence categorized as Good (adherence ≥95% or ≤ 2 missed doses out of 30, or ≤ 3 missed doses out of 60), Fair (adherence 85–94% or 3–5 missed doses out of 30, or 4–9 missed doses out of 60), Poor (adherence <85% or ≥ 6 missed doses out of 30, or ≥ 10 missed doses out of 60); and Follow-up status (alive on ART or ART retention, death, loss to follow-up (LTFU), transfer out).

### 2.4 Data collection and analysis

All relevant documents were gathered and assessed according to a checklist from the designated health facilities during the study period. Data collection was conducted by experienced ART providers under investigator supervision. Completed data collection forms underwent thorough examination for clarity and consistency. Subsequently, data entry and cleaning were performed using Excel, with subsequent analysis conducted using Stata (version 17; Stata Corp, College Station, TX, United States). Descriptive statistics were computed and presented using tables and line graphs. Incidence risk (IR) and incidence rate ratios (IRR) were calculated, with statistical significance set at a *p*-value of less than 0.05.

## 3 Results

### 3.1 Socio-demographic and baseline clinical characteristics of people living with HIV

Between 2017 and 2021, a total of 7,447 individuals living with HIV were registered to begin antiretroviral therapy across various health facilities in the North Shoa Zone of Oromia. Notably, the number of infections exhibited an increasing trend over this period, rising from 4,276 in 2017 to 5,783 in 2021, highlighting a growing public health concern. The overall mean age of the infected individuals was 40.47 years (SD = 13.14), indicating a predominantly middle-aged population affected by the virus.

Demographically, over 60% of those living with HIV in this study were male, reflecting gender disparities in the incidence of the disease. Most participants were receiving first-line treatment regimens, which are crucial for managing HIV effectively and preventing disease progression.

Regarding functional status, the proportion of patients who were bedridden prior to the pandemic was relatively low, suggesting that most individuals were managing their condition effectively. However, the COVID-19 pandemic significantly impacted this demographic. During the pandemic, the percentage of bedridden patients increased to 3.9% in 2020 and 3.8% in 2021, indicating deterioration in health status for some individuals due to the disruptions caused by the pandemic. This trend underscores the urgent need for continued support and monitoring of patients living with HIV, especially during public health crises (Table 1).

**Table 1:** Basic demographic and clinical information of PLHIV at North Shoa Zone stratified by cohort year (2017 to 2021).

Variables	Follow up year				
	2017	2018	2019	2020	2021
Individuals (*n*)	4,276	4,501	4,791	5,672	5,783
Gender (%)					
Male	64.1	64.4	65.2	64.3	64.2
Female	35.9	35.6	34.8	35.7	35.8
Age (%)					
< 16	4.7	5.3	5.0	4.9	4.4
≥ 16	95.3	94.7	95.0	95.1	95.6
Functional status					
Bedridden	0.9	1.2	1.2	3.9	3.8
Ambulatory	2.9	3.0	2.9	2.7	1.9
Working	96.2	95.8	96.0	93.4	94.3
WHO stage (%)					
1	39.8	38.7	38.9	38.0	37.0
2	27.9	27.5	28.2	26.8	28.4
3	29.6	30.8	29.8	31.6	31.4
4	2.7	2.9	3.1	3.6	3.1
Regimen line (%)					
1	94.9	94.4	94.3	93.7	93.4
2	4.3	5.6	5.7	6.26	6.5
3	0	0	0	0.04	0.1

### 3.2 Retention in care

Annual retention rates for people living with HIV from 2017 to 2019 were consistently high, ranging from 0.941 to 0.949. This stability reflects effective management and support systems in place during that period. However, the onset of the COVID-19 pandemic had a dramatic impact on these rates. In 2020, the annual retention rate dropped to 0.837, and it slightly increased to 0.840 in 2021, indicating a substantial decline in retention during the pandemic compared to pre-COVID-19 levels (Table 2).

**Table 2:** Annual retention rate of people living with HIV from 2017 to 2021 at North Shoa Zone, Oromia, Ethiopia.

Follow-up year	Dead	LTFU	TO	Alive on ART	Total PLHIV	Retention rate
2017	41	91	84	4,060	4,276	0.949
2018	60	98	84	4,259	4,501	0.946
2019	58	118	107	4,508	4,791	0.941
2020	237	413	273	4,749	5,672	0.837
2021	230	398	290	4,865	5,783	0.841

LTFU: Lost to follow up; TO: Transfer to out which means transfer to other health facility.

**Figure 1:**
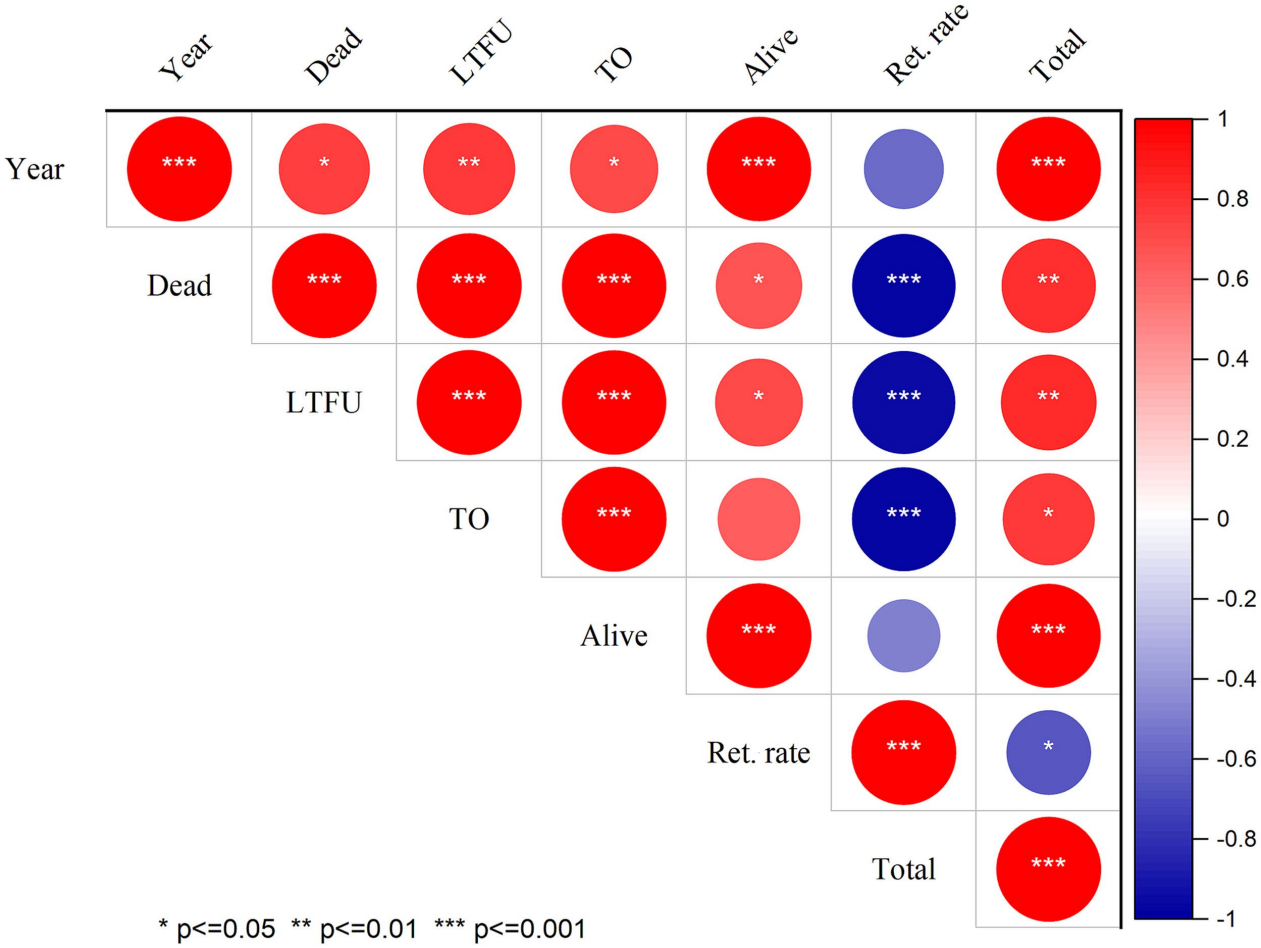
Correlation of follow-up status and retention rate of PLHIV at North Shewa Zone, Oromia, Ethiopia. LTFU: Lost to follow up; TO: Transfer to out which means transfer to other health facility, Ret. rate: Retention rate.

**Figure 2:**
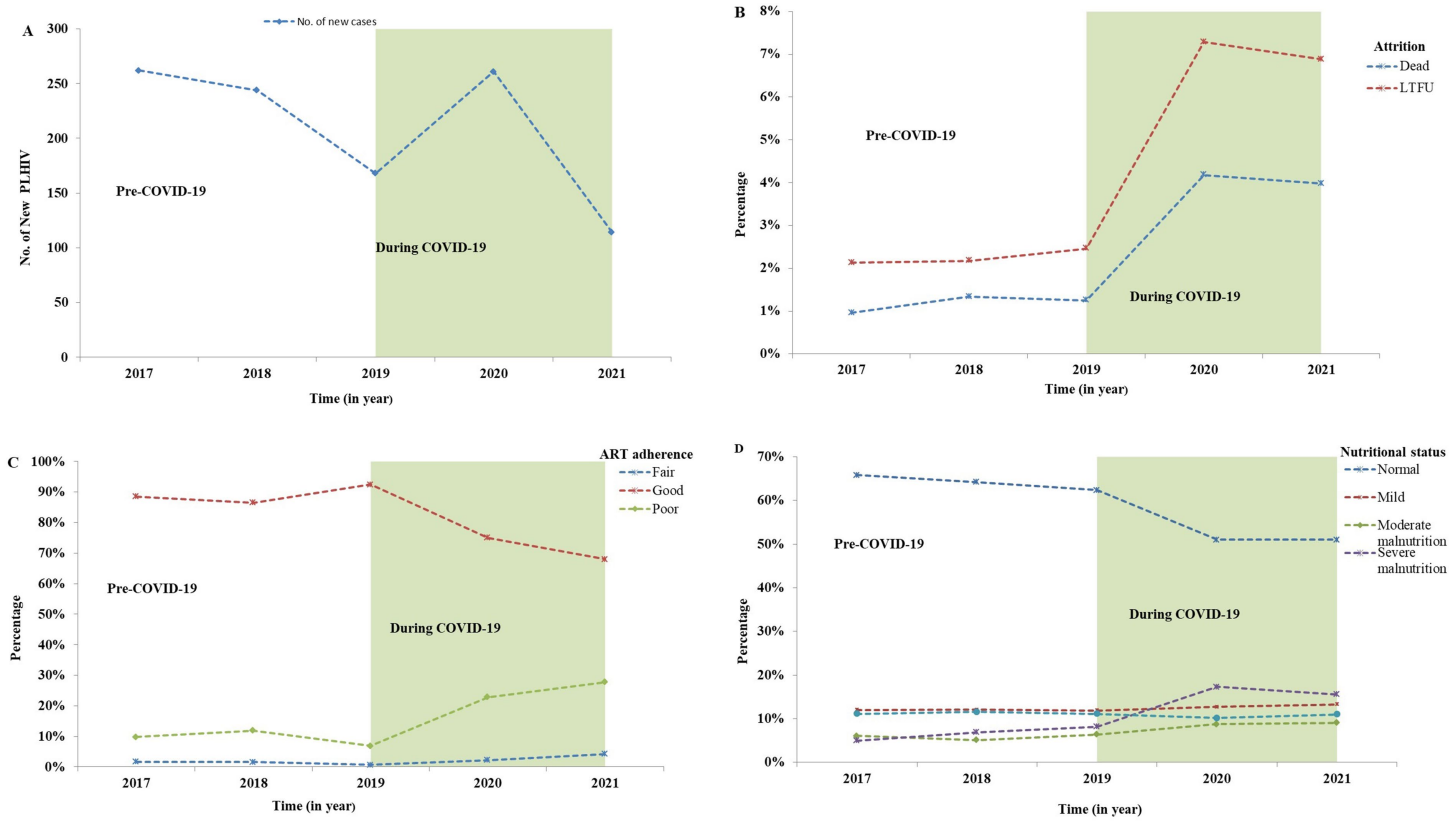
Comparison of the number of new HIV cases, and the percentage of follow-up status, adherence, and nutritional screening of PLHIV in North Shewa Zone, Oromia, Ethiopia from 2017 to 2021. **(A)** Number of new HIV cases, **(B)** Attrition rates (deaths and LTFU) before and during the COVID-19, **(C)** ART adherence categorized as good, fair, and poor, **(D)** Nutritional status of PLHIV categorized as normal, mild, moderate, and severe malnutrition. LTFU: Lost to follow-up; TO: Transfer out (transferred to another health facility).

Moreover, retention rates were significantly negatively correlated with (LTFU) (r: −0.959, *p* ≤ 0.001), death (r: −0.968, *p* ≤ 0.001), and transfer out (r: −0.979, *p* ≤ 0.001). These correlations highlight the interrelated nature of patient retention and adverse outcomes, suggesting that lower retention rates are associated with higher incidences of LTFU, mortality, and transfers. Additionally, a moderate negative correlation was observed between retention rates and follow-up years (r: −0.577, *p* = 0.081), although this correlation was not statistically significant. This finding implies that as the duration of follow-up increased, retention may decline, warranting further investigation into factors influencing long-term patient engagement in care (Figure 1). In addition, the number of new cases starting ART also declined during the COVID-19 compared to the pre-pandemic period. This decline in new ART initiations (Figure 2A) may reflect both the challenges in accessing healthcare services during the pandemic and potential delays in diagnosis, leading to fewer people entering care during this critical time.

### 3.3 Attrition (LTFU and death)

Follow-up discontinuation and HIV-related deaths were key variables in this study. Notably, significant increases in LTFU and deaths were observed during the COVID-19 pandemic compared to the pre-pandemic period. The percentage of both deaths and LTFU rose markedly during the COVID-19 outbreak, as illustrated in Figure 2B.

Specifically, the IRR for LTFU increased significantly during the pandemic, reaching an IRR of 3.00 (95% CI: 2.71–3.33, *p* ≤ 0.001). This indicates that individuals living with HIV were three times more likely to experience LTFU during the pandemic than before. Similarly, mortality rates during COVID-19 were substantially elevated, with an IRR of 3.61 (95% CI: 3.13–4.16, *p* ≤ 0.001) compared to the pre-pandemic period (Table 3). These findings underscore the adverse impact of the pandemic on the retention of individuals in HIV care, highlighting the urgent need for targeted interventions to address the challenges faced by this vulnerable population during such crises.

**Table 3:** Incidence rate and incidence risk ration of basic outcome of people living with HIV at North Shoa Zone stratified by before outbreak of COVID-19 (2017 to 2019) and during COVID-19 (2020 to 2021).

Variables	IR per 100,000 person year		IRR (95% CI)	*p*-value
	Before COVID-19	During COVID-19		
Attrition				
LTFU	1.17	3.5	3.00 (2.71–3.33)	≤ 0.001
Dead	0.56	2.02	3.61 (3.13–4.16)	≤ 0.001
Poor adherence	4.38	9.95	2.27 (2.14–2.40)	≤ 0.001
Nutritional screening				
Mild malnutrition	6.45	6.93	1.07 (1.01–1.14)	0.018
Moderate malnutrition	2.99	4.58	1.53 (1.42–1.65)	≤ 0.001
Severe malnutrition	3.56	8.27	2.32 (2.18–2.47)	≤ 0.001
Overweight	5.04	4.45	0.88 (0.82–0.95)	≤ 0.001

IR: Incidence rate, IRR: Incidence rate ratio, LTFU: Lost to follow up.

### 3.4 ART adherence level

This study compared ART adherence levels before the COVID-19 outbreak to those during the pandemic. The findings revealed a significant increase in poor and fair levels of ART adherence during the COVID-19 outbreak compared to the pre-pandemic period, as shown in Figure 2C.

The IRR for ART adherence levels indicated substantial changes, with fair adherence levels increasing significantly (IRR: 2.39, 95% CI: 2.08–2.76, *p* ≤ 0.001) and poor adherence also rising significantly (IRR: 2.27, 95% CI: 2.14–2.40, *p* ≤ 0.001) during the COVID-19 outbreak compared to the pre-COVID-19 period (Table 3). These results highlight the detrimental effects of the pandemic on adherence to ART, emphasizing the need for enhanced support strategies to maintain adherence levels among individuals living with HIV during challenging times.

### 3.5 Nutritional screening

The study also investigated the nutritional status of individuals living with HIV, comparing data from the pre-COVID-19 period to that during the pandemic. Figure 2D illustrates the classification of nutritional status and the notable changes that occurred during COVID-19, revealing a substantial increase in the number of moderately and severely malnourished individuals during this time.

The IRR further highlighted significant changes in nutritional status during COVID-19. Severe malnutrition increased dramatically (IRR: 2.32, 95% CI: 2.18–2.47, *p* ≤ 0.001), alongside moderate malnutrition (IRR: 1.53, 95% CI: 1.42–1.65, *p* ≤ 0.001) and mild malnutrition (IRR: 1.07, 95% CI: 1.01–1.14, *p* ≤ 0.05). In contrast, there was a 12% reduction in the prevalence of overweight individuals during the pandemic compared to the pre-outbreak period (Table 3). These findings underscore the adverse impact of the pandemic on the nutritional well-being of people living with HIV, highlighting the need for targeted nutritional support during health crises.

## 4 Discussion

This study investigated the follow-up status of people living with HIV over five cohort years, comparing results from before and during the COVID-19 pandemic. The primary focus was on assessing retention in HIV care, attrition rates, levels of ART adherence, and nutritional screening status among individuals living with HIV. The findings of this study provide critical insights into how the pandemic has impacted these key areas of HIV care and are discussed in detail below.

The study revealed that retention rates for HIV care during the COVID-19 pandemic were lower compared to pre-pandemic levels. This decline has a detrimental impact on the survival of people living with HIV, as retention in HIV care is strongly associated with survival from HIV infection. High retention in HIV care predicts favorable survival outcomes (25, 26), whereas low retention is linked to poorer survival rates among individuals with HIV. Additionally, the study suggests that poor retention in HIV care is associated with a lower rate of viral suppression (27–29). It’s crucial for all people living with HIV, even those with suppressed viral loads, to remain engaged in care to prevent the virus from rebounding to detectable levels, which can increase the risk of viral transmission.

Additionally, our study observed a significant increase in the incidence of LTFU during the COVID-19 pandemic compared. This increase in LTFU was found to be significantly associated with retention in HIV care, posing serious challenges to the ongoing efforts to achieve the UNAIDS 95–95-95 targets by 2030 in Ethiopia. Previous studies have similarly reported rising rates of LTFU from HIV care during the pandemic (21, 30, 31). The heightened LTFU undermines broader efforts to ensure that 95% of people living with HIV are aware of their status, as it indicates gaps in the healthcare system that can affect outreach and testing initiatives for those who have not yet been diagnosed. Furthermore, as individuals disengage from care, the percentage receiving sustained antiretroviral therapy declines, directly impacting the second target. This, in turn, affects the third target of achieving viral suppression, as lower adherence to ART can lead to increased viral loads and a higher risk of transmission. According to the UNAIDS 2023 report on progress toward the 95–95-95 targets, Ethiopia achieved 90, 85, and 75% for the respective targets (32). This indicates that Ethiopia needs to intensify efforts to reach these goals by 2030. Therefore, assessing the rate of LTFU is crucial for implementing targeted interventions aimed at restoring normal follow-up among people living with HIV (33), which is essential for meeting the UNAIDS targets and ultimately ending the AIDS epidemic in Ethiopia.

Furthermore, our study identified an elevated incidence of poor ART adherence during the COVID-19 pandemic. This decline in adherence raises concerns, as poor adherence can lead to drug resistance (34), increase the risk of comorbidities, exacerbate existing health conditions, and result in higher healthcare costs and mortality rates associated with HIV/AIDS (35). Additionally, studies have shown a clear association between poor adherence to ART and unsuppressed viral loads, which complicates treatment efforts (36). These factors pose significant barriers to achieving the UNAIDS 95–95-95 targets, particularly the second and third targets focused on sustained treatment and viral suppression.

We also assessed the nutritional status of people living with HIV during the pandemic through nutritional screening. Our findings revealed a significant increase in the incidence of moderate and severe malnutrition compared to pre-pandemic levels. This deterioration in nutritional health may adversely affect the survival of individuals living with HIV, as maintaining a good nutritional status is crucial for supporting the immune system and overall well-being (37). Proper nutrition enhances the effectiveness of HIV treatment by promoting weight maintenance and optimizing medication absorption. Conversely, malnutrition can reduce ART efficacy and increase HIV-related morbidity and mortality (38, 39). The heightened risk of opportunistic infections (OIs) associated with undernutrition further complicates care in a resource-limited setting like Ethiopia (40). These nutritional challenges directly impact the achievement of the UNAIDS targets, as they impede retention in care and treatment adherence. Finally, our study identified a notable rise in mortality among people living with HIV during the COVID-19 pandemic compared to pre-pandemic levels. This increase can likely be attributed to heightened rates of loss to follow-up, uncontrolled viral loads, and inadequate adherence to treatment. Previous research has established a correlation between suboptimal adherence and increased mortality rates (41, 42). The compounded effects of LTFU, poor ART adherence, and malnutrition underscore the urgent need for comprehensive strategies that address these interconnected issues. Failure to tackle these challenges jeopardizes progress toward the UNAIDS 95–95-95 targets. Specifically, increased mortality and LTFU hinder efforts to ensure that 95% of people living with HIV know their status and receive sustained treatment, ultimately affecting the percentage achieving viral suppression. Addressing these issues is essential not only for improving health outcomes for people living with HIV but also for ensuring the long-term sustainability of HIV care and treatment programs in Ethiopia, particularly in the face of ongoing public health emergencies.

However, several limitations of this study should be acknowledged. First, the retrospective design relies on the quality and completeness of available data, which may have affected the results due to missing or incomplete records. Second, the focus on selected health facilities in the North Shoa Zone of Oromia, Ethiopia, limits the generalizability of findings to other regions or countries. Third, the impact of the COVID-19 pandemic presents a challenge, as isolating its direct effects from confounding factors like socio-economic conditions and healthcare infrastructure difficulties is complex. Fourth, although nutritional status was assessed, reliance on existing records may not fully capture the nuanced challenges individuals faced during this period. Fifth, the study did not consider external influences, such as economic stressors, transportation issues, or changes in healthcare policies that may have contributed to follow-up discontinuation. Lastly, the analysis was confined to comparing pre-COVID-19 (2017–2019) and during-COVID-19 (2020–2021) periods, without exploring potential long-term post-pandemic effects on HIV care retention and outcomes.

## 5 Conclusion

This study identified significant healthcare disruptions among people living with HIV during the COVID-19 pandemic. We observed a notable increase in the incidence of LTFU, poor adherence to treatment, moderate and severe malnutrition, and mortality compared to the pre-COVID-19 period. Additionally, retention rates in HIV care have markedly declined during the pandemic. These interruptions not only jeopardize the survival rates of individuals living with HIV but also contribute to increased transmission of the virus, exacerbating the disease burden in Ethiopia. Our analysis highlights critical deficiencies in the delivery of HIV care during this crisis and underscores the need for proactive measures to address these challenges in future public health emergencies. It is essential for both governmental and non-governmental organizations to prioritize the needs of people living with HIV in emergency responses, recognizing their heightened vulnerability to health-related crises. By doing so, these efforts can better support this population and work towards achieving the UNAIDS 95–95-95 targets by 2030.

## Data Availability

The raw data supporting the conclusions of this article will be made available by the authors, without undue reservation.
